# A useful treatment for patients with advanced mixed-type small cell neuroendocrine carcinoma of the prostate: A case report

**DOI:** 10.3892/ol.2013.1136

**Published:** 2013-01-15

**Authors:** KEI-ICHIRO UEMURA, GO NAKAGAWA, KATSUAKI CHIKUI, FUKUKO MORIYA, MAKOTO NAKIRI, TOKUMASA HAYASHI, SHIGETAKA SUEKANE, KEI MATSUOKA

**Affiliations:** 1 Departments of Urology, Kurume University School of Medicine, Kurume, Fukuoka 830-0011, Japan; 2 Pathology, Kurume University School of Medicine, Kurume, Fukuoka 830-0011, Japan

**Keywords:** prostatic small cell neuroendocrine carcinoma, external-beam radiotherapy with intra-arterial chemotherapy, ischuria, locally advanced tumor

## Abstract

Treating extended prostatic small cell neuroendocrine carcinoma (PSCNC) is extremely difficult and no standard treatment has yet been established. We experienced a case of advanced mixed-type PSCNC in which the patient achieved long-term survival and local control following combined therapy. Locally advanced PSCNC causing lower urinary obstruction was detected during androgen-ablation therapy for stage D2 mixed adenocarcinoma PSCNC. The patient was treated with intra-arterial infusion chemotherapy using a reservoir system and external-beam radiotherapy (EBRT) to the whole pelvis and local tumor. After chemoradiotherapy, the patient’s lower urinary obstruction was reduced and did not return during the remaining 40 months of the patient’s life. The patient survived for 70 months following the start of the androgen-ablation therapy. The present study reports a useful treatment for advanced mixed-type PSCNC, androgen-ablation therapy and chemoradiotherapy. The present results also suggest that the prognostic factors for advanced mixed-type PSCNC are the sensitivity of the conventional adenocarcinoma to androgen-ablation therapy, degree of metastasis and extent of the small cell neuroendocrine carcinoma component.

## Introduction

Prostatic small cell neuroendocrine carcinoma (PSCNC) is an extremely rare type of prostate cancer that accounts for 0.5–2% of prostatic primary tumors (1,2). In almost all cases, the tumor is detected at an advanced stage since prostate-specific antigen (PSA) is unreliable for detecting PSCNC. The present case was detected during androgen-ablation therapy, at which point the patient exhibited local symptoms of ischuria and gross hematuria, as well as a slightly increased PSA value. The usual treatment for PSCNC is systemic chemotherapy, with or without radiotherapy, involving a similar regimen to that used for pulmonary small cell carcinoma (PSCC). In the present case, following the androgen-ablation therapy, only a locally advanced tumor and right obturator lymph node metastasis remained; therefore, it was possible to perform external-beam radiotherapy (EBRT) and intra-arterial chemotherapy. This treatment was effective against the local tumor and reduced the lower urinary tract symptoms, such as gross hematuria and ischuria. The above-mentioned combination therapy maintained the patient’s quality of life for a relatively long period and contributed to extending the patient’s survival. Accordingly, this therapy should be recommended as a treatment option for cases of locally advanced PSCNC involving lower urinary obstruction.

This study was approved by the Ethics Committee of Kurume University (Kurume, Fukuoka, Japan). Written informed consent was obtained from the patient’s family.

## Case report

**Figure 1:**
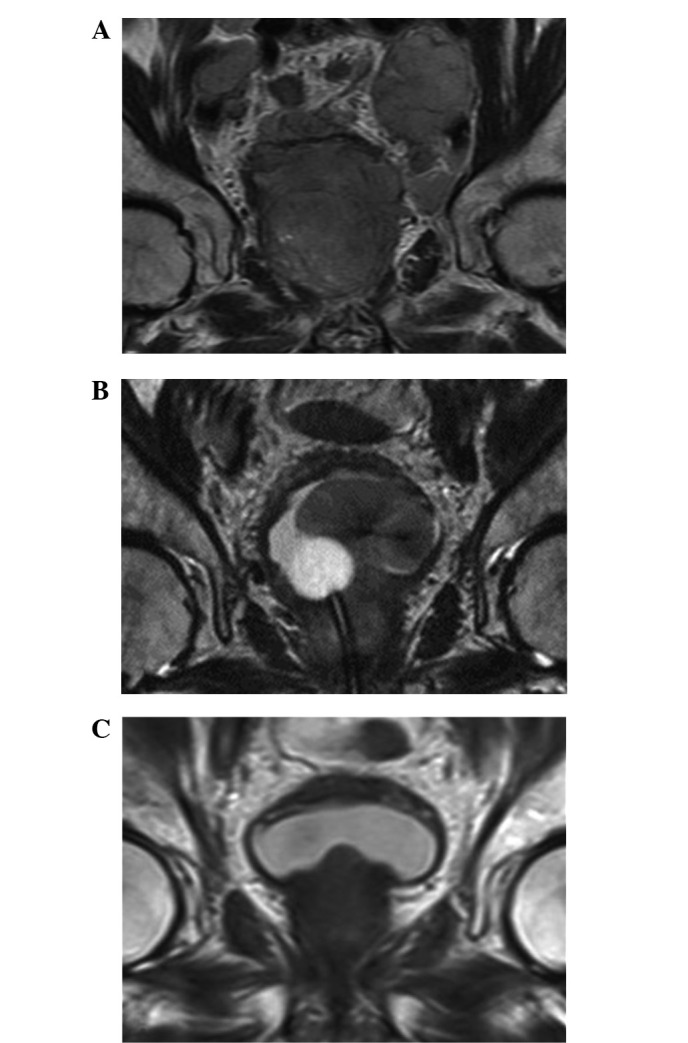
(A) Pelvic MRI obtained prior to the androgen-ablation therapy demon strating the presence of an invasive prostatic tumor and numerous large metastatic LN lesions. (B) Pelvic MRI following the androgen-ablation therapy demonstrating a locally advanced prostatic tumor, which projected into the bladder and had reduced LN swelling. (C) Pelvic MRI following the combination treatment involving EBRT and intra-arterial infusion chemotherapy demonstrating that the prostatic tumor had markedly diminished. LN, lymph node; EBRT, external-beam radiotherapy.

**Figure 2:**
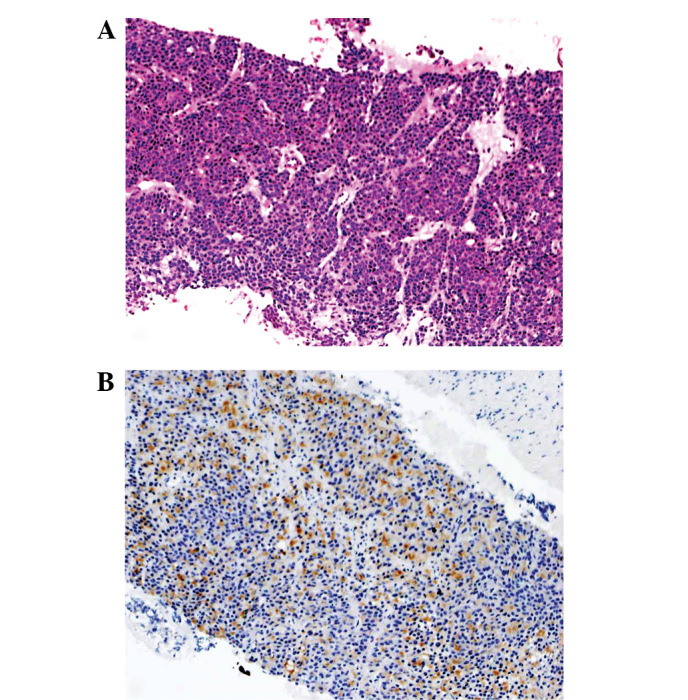
(A) Transrectal biopsy produced a diagnosis of poorly differentiated adenocarcinoma with small cell NE carcinoma. HE staining produced an initial diagnosis of Gleason pattern 5b poorly differentiated adenocarcinoma (magnification, ×100). (B) PSA staining revealed that PSA-positive and -negative cells were intermixed in the biopsy sample (magnification, ×100). HE, hematoxylin and eosin; NE, neuroendocrine; PSA, prostate-specific antigen.

**Figure 3:**
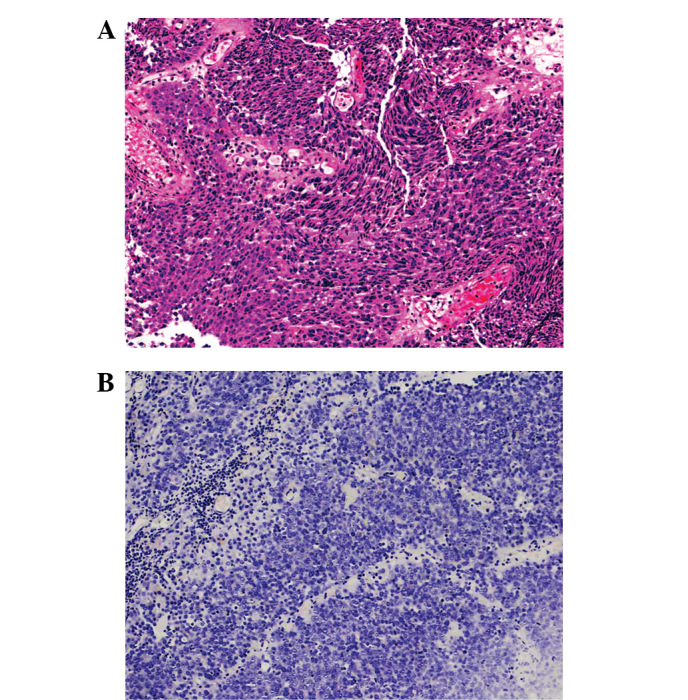
(A) Transurethral biopsy produced a diagnosis of small cell NE carcinoma. HE staining revealed that these carcinoma cells had similar morphological features to the transrectal biopsy tissue carcinoma cells (magnification, ×100). (B) None of the tumor cells were positively stained for PSA (magnification, ×100). NE, neuroendocrine; HE, hematoxylin and eosin; PSA, prostate-specific antigen.

A 75-year-old male Japanese patient was admitted to hospital with a high PSA value (95 ng/ml) and right leg edema. Abdominal ultrasonography, transrectal ultra-sonography, computed tomography (CT), magnetic resonance imaging (MRI; Fig. 1A) and transrectal biopsy (Fig. 2A) were performed. The patient was diagnosed with prostate cancer (poorly differentiated adenocarcinoma, Gleason score: 5+5=10, T4N1M1a stage D2) and received androgen-ablation therapy involving goserelin acetate (10.8 mg/3 months) and bicalutamide (80 mg/day) in October 2005. Subsequently, the patient’s PSA value fell, reaching 0.032 ng/ml in February 2007. However, the patient gradually became aware of dysuria and gross hematuria and his PSA value gradually began to rise again. Eventually, the patient developed ischuria so a urethral catheter was inserted to relieve the lower urinary obstruction in September 2007. In October 2007, MRI (Fig. 1B) detected a large tumor extending from the prostate to the bladder and the patient’s PSA value was 0.151 ng/ml at that time. A transurethral biopsy was performed (Fig. 3A) and the patient was diagnosed with PSCNC that occurred following the androgen-ablation therapy. An immunohistochemical examination of the biopsy tissue revealed that the tumor cells were negative for PSA (Fig. 3B), neuron-specific enolase (NSE), chromogranin A and CD56, but positive for synaptophysin. Subsequently, an additional immunohistochemical examination of the transrectal biopsy tissue was performed, which identified carcinoma cells showing the same results as the transurethral biopsy tissue cells with the exception of PSA, which revealed the intermingling of PSA positive and negative cells (Fig. 2B). The immuno histochemical findings are presented in Table I. Chest-to-pelvis CT scanning detected a large prostatic tumor and a right obturator lymph node (LN) swelling of 2.5 cm, while bone scintigraphy did not detect any abnormal lesions. Consequently, the patient was diagnosed with prostate cancer and the local occurrence of PSCNC during androgen-ablation therapy for advanced mixed-type PSCNC.

The decision was made to administer EBRT and an intra-arterial infusion of cisplatin (CDDP; 20 mg/m^2^; Days 1–5) and ifosfamide (IFM; 1.2 mg/m^2^; Days 1–3) as treatments for the local tumor and ischuria (regimen cycle, 21 days). An indwelling catheter was placed in the bilateral internal iliac artery and a pump was placed in a subcutaneous pocket in November 2007. At that time, the patient’s serum PSA level was 0.144 ng/ml.

From December 2007, the patient received 3 courses of intra-arterial chemotherapy and also underwent EBRT of the whole pelvic cavity and the local tumor with the swollen lymph node at a total dose of 67 Gy. In the present case, no serious side-effects that posed a risk to the continuation of the intra-arterial infusion chemotherapy were observed. The tumor volume was reduced in MRI (Fig. 1C) and therefore, the urethral catheter was removed in April 2008 and the patient had no urinary symptoms. The patient’s PSA value had decreased to 0.015 ng/ml by June 2008.

However, the patient’s PSA value gradually increased and bone and LN metastasis occurred, although radiographically, the volume of the local mass remained stable. The patient succumbed in August 2011 at 70 months after the start of the androgen-ablation therapy. At the time of the patient’s mortality, he had not required a urethral catheter for 40 months, although he lost uresiesthesia due to the wide-ranging spinal bone metastasis and developed incontinence. No autopsy was performed since we were unable to obtain the approval of the patient’s family.

## Discussion

Extra-pulmonary small cell carcinoma (EPSCC) accounts for ∼0.1% of all cancers (3) and only 2.5–5% of small cell carcinomas occur outside of the lungs (4,5). *De novo* prostate cancer involving small cell carcinoma is extremely rare in patients that are diagnosed by biopsy, with an incidence of 0.5–2% (1,2). However, in a report examining auto psied prostatic cancer cases, small cell carcinoma showed an incidence of 10–20% (6). In addition, Haider *et al*(3) reported that the prostate and neck of the uterus were the two most common organs from which EPSCC originates and that the two most commonly affected sites were the gastrointestinal tract and the genitourinary tract. From a review of the literature, there appear to be three patterns of PSCNC; 35.4% of cases exhibited pure small cell neuroendocrine (NE) carcinoma, 17.7% of cases involved mixed adenocarcinoma and 46.9% of cases demonstrated recurrence involving small cell NE carcinoma that had differentiated from conventional adenocarcinoma during androgen-ablation therapy (7,8). Thus, we named these types pure-type PSCNC, mixed-type PSCNC and differentiated-type PSCNC, respectively.

The prognosis of PSCNC is extremely poor (9). With regard to the prognosis of primary PSCNC, Deorah *et al*(10) reported that the median survival periods for patients with the local/regional disease (a primary tumor and regional lymph node metastasis only) and metastatic disease were 15 and 7 months, respectively, and the 12, 24, 36, 48 and 60-month survival rates were 47.9, 27.5, 19, 17 and 14.3%, respectively. Furthermore, mixed adenocarcinoma-type patients live 3–5 months longer than patients with pure small cell carcinoma (7,8).

Several reports have examined the predictors of a poor prognosis in mixed-type PSCNC. It was reported that the presence of the mixed-type adenocarcinoma was predictive of the degree of metastatic disease and a total lack of hormone responsiveness (11). Deorah *et al*(10) reported that concomitant well-to-moderately differentiated adeno carcinomas were associated with an improved prognosis in small cell carcinoma of the prostate.

With regard to the histological findings of PSCNC, it is important to note that PSCNC cells exhibit a similar morphology to PSCC and Gleason pattern 5b prostate adenocarcinoma cells. Consequently, small cell NE carcinoma is often mistaken for Gleason pattern 5b adenocarcinoma, as occurred in the present case (12).

Immunohistochemical staining is often used to detect PSA, NSE, ProGRP, synaptophysin and chromogranin A in PSCNC, although other markers have also been reported to be effective. Yao *et al*(12) reported that PSCNC exhibits a positive PSA percentage of 17% and suggested that PSA, thyroid transcription factor-1 (TTF-1) and CD56 were useful for distinguishing PSCNC from Gleason pattern 5b adenocarcinoma. The authors also reported that numerous PSCNC cells were positive for bombesin/GRP, c-kit, bcl-2 and EGFR. The majority (80%) of small cell carcinoma cells were positive for at least one neuroendocrine marker. However, negative immunostaining for these markers does not exclude small cell carcinoma.

The most curative treatment for PSCNC is radical prosta tectomy, although it is only indicated for early stage and limited tumors (1). However, PSCNC is an extemely aggressive disease and the majority of cases exhibit metastatic lesions and a large mass at diagnosis. Thus, there is no established treatment for PSCNC. With regard to the treatment for PSCC, the most effective method for limited PSCC is chemoradiotherapy using systemic chemotherapy involving CDDP and etoposide in combination with EBRT involving a total dose of 45 Gy and the most effective method for extensive-stage PSCC is four to six cycles of systemic chemotherapy of CDDP and etoposide (non-Asian patients) or CDDP and irinotecan (Asian patients). Moreover, additional thorax radiotherapy has improved the prognosis of advanced PSCC in which extensive-stage non-thorax cases achieved CR and those with intra-thorax disease achieved PR following three courses with the systemic chemotherapy of CDDP and etoposide (13). Accordingly, in the majority of cases, advanced PSCNC is treated with systemic chemotherapy involving the CDDP/etoposide regimen with or without EBRT as well as PSCC. In the present case, intra-arterial chemotherapy was selected in order to increase the efficacy of the treatment against the local lesion and reduce the frequency/grade of the side-effects (14). The CDDP/IFM regimen was selected and considered to be an appropriate choice as it was an effective carcinostatic and was the most suitable treatment option of those that were available for prostate cancer under the Japanese national healthcare insurance scheme at the time of the patient’s treatment (14). With regard to prophylactic cranial irradiation (PCI), EPSCC is not usually indicated for PCI since the frequency of brain metastasis in EPSCC is lower than that in PSCC (15). A small number of reports have revealed that PSCNC is not suitable for routine PCI (16), although another report observed that the incidence of brain metastasis from PSCNC was ∼10% (11). Vashchenko and Abrahamsson (17) reported several new useful treatment agents for NE differentiation in prostate cancer, including somatostatin analogs, serotonin antagonists, bombesin antagonists and inflammatory cytokines, such as interleukin-6. These new agents are expected to prolong the survival of PSCNC patients. The relaxin (RLN) receptor RXFP1 is also a potential target for PSCNC treatments. Feng *et al*(18) reported that the suppression of RLN/RXFP1 produced a significant reduction in tumor volume, which was associated with decreased cell proliferation and increased apoptosis in PC3 prostate cancer cell lines (the group to which small cell NE carcinoma belongs).

PSCNC is sensitive to chemoradiotherapy, which is able to achieve local control and prolong survival, similar to its effects in PSCC. This suggests that combined therapy involving androgen-ablation therapy and chemoradiotherapy is beneficial for advanced mixed-type PSCNC. In addition, we suggest that the prognostic factors for advanced mixed-type PSCNC are as follows: the sensitivity of the conventional adenocarcinoma to androgen-ablation therapy, degree of metastasis (the number of metastatic organs and the size of any masses) and extent of the small cell NE carcinoma component.

## Figures and Tables

**Table I:** Details of immunohistochemical findings.

Markers	Before androgen-ablation	Subsequent to androgen-ablation
PSA	+/−	−
CgA	−	−
NSE	−	−
Syn	+	+
CD56	−	−

PSA, prostate-specific antigen; CgA, chromogranin-A; Syn, synaptophysin;

+, extensively positive;

+/−, focally positive;

−, negative.
